# Vertebral Fractures

**Published:** 1893-05

**Authors:** Joseph Kurtz

**Affiliations:** Los Angeles, Cal.


### VERTEBRAL FRACTURES.

By JOSEPH KURTZ, M. D., Los Angeles, Cal.

The symptoms of vertebral fractures consist in :

1. Pain ; sometimes a sudden, sharp pain at the seat of the fracture, connected with a sensation of something giving way, and the patient may exclaim, " My back is broken ; " occasionally the pain is not very severe.

2. Deformities ; in fractures of the spinous processes there is always a lateral displacement of the outer fragments, which can be easily seen and felt. The fragments of the lateral processes and arches do not give rise to much deformity, though displaced. But when the bodies are fractured with displacement, we generally notice the characteristic elevation of their spinous processes, forming the kyphosis, which may be aggravated by displacement of a whole vertebra.

3. Paralysis ; complete or incomplete, sensory and motor, which may affect all those parts supplied by nerves arising from below the seat of the injury ; therefore, the higher the fracture, the greater the area of paralysis. The most dangerous is the respiratory paralysis, which results from fracture of the upper cervicals. In fractures of the spinous processes, the medulla usually escapes injury, and no paralysis follows ; the fragments of the other processes and arches generally disturb the medulla more or less, and, accordingly, cause either motor paralysis and hyperesthesia, or only paresis, or severe reflex movements ; or we may observe only the symptoms of concussion. The fractures of the bodies, which so often crush or compress the medulla, give rise to the severest nervous symptoms. Sometimes complete paralysis of all the parts below the seat of injury may set in at once, affecting the bowels and the bladder ; trophic disturbances soon follow, with decubitus, and death is very often desirable to end the scene.

As the symptoms and also the prognosis vary in the different vertebral fractures, I shall now consider the symptoms of these injuries in the various segments of the spinal column :

(*a.*) The upper two cervicals usually break at the posterior arch of the atlas and through the odontoid process of the second. While it was generally understood that these fractures always dislocate the head and atlas forward, and thereby sever the medulla, it was recently proved by Gurlt, of Berlin, that such dislocations do not always result. But even cases without much displacement generally end fatally, partially from respiratory paralysis and partially from myelitis. These fractures are usually easily diagnosed by palpation, external as well as through the mouth, where the prominent atlas may be felt in the pharyngeal wall in a line with the septum nares.

(*b.*) Fractures of the rest of the cervical vertebræ and down to the second dorsal are more frequent, and are particularly dangerous by their injury to the phrenic nerve, which is, as a rule, fatal. In all cervical fractures, if they do not end in death soon, we notice a peculiar wave of temperature, perhaps from 100° in the morning rising to 103° in the night; also hyperemia of the face and anemia of the trunk, disturbance of deglutition, speech, and respiration. Priapism is considered a cervical symptom and paralysis according to seat and extent of injury. The mortality in the fractures so far considered amounts to about eighty-five per cent.; the fifteen per cent. who survive frequently suffer with paralysis or paresis if the injury was severe.

(*c.*) Fractures of the bodies and arches of the other dorsals are characterized by the prominence of the respective spinous processes and nervous symptoms in the lower extremities and abdomen, as paraplegia, tympanites, paralysis of rectum and bladder, followed by cystitis and decubitus. About eighty per cent. die, and the twenty per cent. who recover rarely enjoy good health.

(*d.*) Fractures of the lumbar bodies and arches offer relatively the best prognosis, particularly those of the lower vertebræ, on account of their great strength and the absence of the medulla. In the upper two, the mortality is still about forty per cent., with sixty per cent. of imperfect recoveries.—*Southern California Practitioner*, January, 1893.

---

### BOVINE CONSUMPTION AND THE MILK SUPPLY.

Dr. E. F. Brush has paid special attention to the subject of tuberculosis as it appears in cattle, and the danger of milk from tuberculous cows. He states that this disease is much more common in cattle than is generally believed. As the temperature is not sensibly raised, the cow lives a long time, in apparently good health. The milk is not necessarily or at all times contaminated with tubercle bacilli, but there is not at any time a certainty that it is not so contaminated. Consequently, the child fed on such milk is in about the same danger as an infant that suckles from a syphilitic wet-nurse—infection may or may not occur. The risk, such as it is, should be clearly understood, and those who choose to take their chances do so knowingly. It is to be hoped that these investigations may lead to a reform in the care of milch cattle, whose accommodations are often of the most unsanitary description. As in the human subject, cleanliness, good ventilation, good food, and good hygiene generally, are the best preventatives of bovine tuberculosis.—*Food*, February, 1893.

---

### HEART FAILURE.

Prof. Alfred L. Loomis, M. D., LL.D., read recently before the American Climatological Association a paper on Heart Failure.

He includes all heart failure in three classes :

1. Those in which the heart has for a long time been called upon to perform an abnormal amount of work, as in valvular or arterial disease.

2. Those in which obstructive changes in the coronary vessels markedly diminish the nutritive supply of the cardiac muscle.

3. Those in which toxic influences act directly upon the nutrition of the cardiac muscle, or to interfere with the cardiac nerve supply as to lessen cardiac resistance.

He concludes with this excellent advice, in his summary of his conclusions, as to the lessons taught by the facts demonstrated. He says :

"However we may explain it, clinical observation teaches that some chronic and many acute infections so diminish heart power that sudden heart-failure occurs in hearts that previous to this infection were of normal integrity. It then becomes of the utmost importance, in all toxic conditions, to watch for the first indications of cardiac weakness. On this principle Stokes based his great rule for the use of alcoholic stimulants in the treatment of typhoid fever, when he directed, "that in every case of fever, if the first sound of the heart became indistinct, stimulants should immediately be given in sufficient quantities to restore the heart tone." It is on this principle, also, that strychnia upholds an alcoholic heart in pneumonia, by restoring or increasing its nerve supply. A rule which for a long time has governed me in all toxic conditions is, not to wait for signs of commencing heart-failure, but to begin the administration of alcohol, strychnia, and other heart tonics early, and thus, if possible, save my patients from fatal heart-failure.

"A review of the cases which I have presented makes it evident that the term heart-failure is misleading and should be abandoned, for, in most instances, it does not express the pathological state. It is equally evident that the term "death by heart-failure" is often used to cover the ignorance of the medical attendants."—*Medical Age*, March, 1893.

---

### THE PRESENT POSITION OF GALL-BLADDER SURGERY.

Czerny, after a general consideration of gall-bladder surgery, presents the following conclusions:

1. Gall-stones require operation, if they cause frequently repeated or lasting trouble.

2. Empyema of the gall-bladder imperatively demands operation, as does hydrops, if it gives serious trouble.

3. The typical operation for gall-stones consists in incision, removal of the stones, and suture of the gall-bladder; in this, however, the abdominal wound should be drained for a short time.

4. If the cystic duct is closed, if the gall-bladder is the seat of inflammation, or the contents are greatly altered, then a temporary gall-bladder fistula must be made.

5. Extirpation of the gall-bladder is indicated only in cases of severe inflammatory carcinomatous involvement.

6. When the ductus choledochus is closed, the operation is absolutely indicated, if the strength of the patient will permit. If one does not succeed in removing the stone or obstruction, then it is recommended to produce a fistula between the gall-bladder and duodenum.

7. The best incision for gall-bladder operations is an ⌋ shaped cut; the vertical limb lies in the linea alba, and the horizontal part runs towards the right, just below the level of the umbilicus.

8. The danger to life of gall-stone operations will be probably less than in operations for vesical calculus.—*Epitome of Medicine.*

